# Multi modal hierarchical reinforcement learning framework for dynamic sports sponsorship optimization

**DOI:** 10.1038/s41598-025-27915-9

**Published:** 2025-12-18

**Authors:** Qiyang Yu

**Affiliations:** School of Economics and Management, Chengdu Sport University, Chengdu, 641418 Sichuan China

**Keywords:** Reinforcement learning, Sports sponsorship, Multi-modal data, Real time optimization, Audience engagement, Engineering, Mathematics and computing

## Abstract

This paper presents a novel approach to optimizing sports sponsorship strategies by integrating reinforcement learning (RL) with a multi-modal hierarchical framework, enhancing real-time decision-making using diverse data sources such as computer vision, natural language processing, and graph neural networks (GNNs). The system utilizes RL to dynamically optimize sponsorship strategies across strategic, tactical, and operational levels. Using the Meta-Soft Actor-Critic (Meta-SAC) algorithm, it adapts to real-time data streams, including social media sentiment, event footage, and stakeholder interactions. Our system demonstrates a 25–35% improvement in ROI, a 20–30% increase in brand exposure, and a 15–25% rise in audience engagement compared to conventional strategies. The proposed RL-driven, multi-modal framework significantly outperforms traditional methods, providing scalable, adaptive solutions for optimizing sports sponsorship effectiveness.

## Introduction

In the contemporary sports industry, sponsorship has evolved into one of the most significant pillars of marketing strategy. Brands increasingly view sports sponsorships as crucial avenues for enhancing visibility, fostering consumer engagement, and driving revenue growth. The global sports sponsorship market is projected to exceed d $70 billion by 2027, reflecting its immense importance in modern marketing^1^. However, despite the substantial investments brands make in sports partnerships, optimizing the effectiveness of sponsorship strategies remains a complex challenge. Traditional sponsorship evaluation methods, which often rely on post-event surveys or simple metrics like TV viewership, fail to capture the multifaceted nature of sponsorship success^2,3^. Moreover, these methods cannot adapt to real-time changes, missing opportunities to adjust strategies dynamically during events^4^.

Recently, digital platforms and social media have become even more prevalent, and this has added another level of complexity to sports sponsorship. Now brands seek the visibility created at a live event, along with insights into audience sentiment and engagement, and the overall impact on investments. Now we have a good amount of data in an unstructured form like photos, videos, and textual content available over social media platforms, online content and live interactions which cannot be handled by a classical modeling^4,5^. To overcome the challenge, there is an urgent need for sophisticated systems capable of handling data from multiple sources and making timely information valuable in sponsorship decisions. Reinforcement Learning (RL) has witnessed considerable popularity in the decision-making arena from the perspective of modeling a dynamic environment and optimizing the decision strategies through successive interactions with the system^6^. When it comes to sports sponsorship, RL has a unique advantage. It can adjust and improve strategies based on incoming data. Rethink what data-driven can do for you and optimise sponsorship campaigns almost in real-time! Though possible, not many research work has integrated various multi-modal data, such as visual, textual, and graph, into a cohesive framework for optimizing sports sponsorship utilizing RL.

This paper presents a novel approach that integrates reinforcement learning with a hierarchical, multi-modal framework to optimize sports sponsorship. Leveraging technologies such as computer vision, natural language processing (NLP), and graph neural networks (GNNs), our system enhances decision-making across strategic, tactical, and operational levels. It adapts to real-time data, improving key metrics like Return on Investment (ROI), brand exposure, and audience engagement. To our knowledge, this is the first comprehensive RL-based approach that incorporates multi-modal data, such as social media sentiment and visual brand exposure, for sports sponsorship optimization. Our system outperforms traditional methods like rule-based and heuristic approaches by offering dynamic, scalable, data-driven decision-making. The key contributions are:

- A multi-modal RL framework that integrates computer vision, NLP, and GNNs to optimize sponsorship effectiveness.
- The use of the Meta-Soft Actor-Critic (Meta-SAC) algorithm for efficient exploration and exploitation.
- A multi-agent system to optimize interactions among brands, event organizers, and the audience.

Our method shows a 25–35% improvement in ROI over traditional strategies, demonstrating its practical applicability in real-world sports sponsorship management. The rest of this paper is structured as follows. “Related work” reviews related work. “Methodology” presents the proposed methodology. “Experiments and results” describes experiments and results. “Discussion” discusses findings, and “Conclusion” concludes the study and outlines future work.

## Related work

Reinforcement Learning (RL) has emerged as a powerful paradigm for dynamic optimization, finding increasing application in complex systems ranging from smart grids to autonomous vehicles^7–9^. The integration of RL, particularly Deep Reinforcement Learning (DRL), within a multi-modal hierarchical framework offers a promising approach for enhancing sports sponsorship effectiveness through dynamic optimization^10–12^.

The core problem of dynamic optimization in sports sponsorship involves maximizing return on investment by adaptively adjusting sponsorship strategies in response to real-time data and evolving conditions^1,2^. Traditional optimization methods often struggle with the inherent complexity, uncertainty, and competing objectives in such dynamic environments^10,13^. RL, conversely, is specifically designed to tackle sequential decision-making problems by allowing an agent to learn optimal policies through interaction with an environment, receiving rewards or penalties for its actions^3,14,15^.

### Reinforcement learning fundamentals and architectures

RL algorithms aim to optimize decision-making by leveraging interaction samples with an environment and potentially delayed feedback^14,16^. Unlike supervised learning, which relies on exhaustive, one-shot reward signals, RL addresses sequential decision-making with sampled, evaluative, and delayed feedback^14^. This makes RL a suitable candidate for dynamic problems like sports sponsorship optimization. The spectrum of RL algorithms includes model-free approaches like Q-learning and SARSA, which learn directly from experience without an explicit environment model, and model-based approaches, which learn a model of the environment to plan and make decisions^4,7,14^. Deep Reinforcement Learning (DRL) combines deep neural networks with RL, enabling it to handle high-dimensional state and action spaces, which are characteristic of real-world problems such as dynamic traffic management, energy systems, and robotics^3,4,17^. For instance, Deep Q-Networks (DQN) utilize neural networks to approximate the Q-function, which estimates expected future rewards for state-action pairs^7^. Variants like Dueling DQN and Double DQN address limitations such as overestimation and improve learning stability^18^. Proximal Policy Optimization (PPO) is another DRL algorithm designed for stability and efficiency in policy-based methods, updating policies to avoid drastic changes while maximizing rewards^3,19^.

### Multi-modal data integration

The multi-modal aspect refers to the integration of diverse data sources to provide a more comprehensive understanding of the environment and improve decision-making^20,21^. In sports sponsorship, multi-modal data could include audience demographics, social media engagement, brand exposure metrics, athlete performance data, economic indicators, and real-time event data^5,22^. Integrating such heterogeneous data is crucial for capturing the multifaceted nature of sponsorship effectiveness. For example, research on sentiment-aware multi-modal dialogue policy learning highlights the benefits of incorporating data beyond text, such as images, to better understand user behavior^23^. Similarly, physical activity recognition systems leverage multi-modal time-series data like heart rate, speed, and distance, optimized through DRL, to predict activities^17,24^.

Unsupervised multi-view representation learning, as exemplified by frameworks combining Deep Canonical Correlation Analysis (DCCA) and Generalized Canonical Correlation Analysis (GCCA), can learn meaningful shared representations from multiple data views, addressing discrepancies and integrating complementary information^21^. This approach can be vital in aggregating disparate sponsorship data for a unified representation feeding into an RL agent.

### Hierarchical reinforcement learning frameworks

The hierarchical component is essential for managing the complexity of dynamic optimization problems by decomposing large tasks into smaller, more manageable sub-tasks^10,12,25,26^. Hierarchical Reinforcement Learning (HRL) allows agents to operate at different levels of abstraction, with high-level policies making strategic decisions and low-level policies handling tactical actions^10^. For instance, a high-level policy in sports sponsorship might decide on the overall budget allocation across different sports or events, while a low-level policy could optimize ad placements or social media campaigns within a specific event.

HRL has been applied in various complex domains, such as multi-agent cooperative maneuver interception in dynamic environments, where it decomposes tasks into dynamic allocation and distributed control stages^12^. In multi-agent systems, HRL can accelerate the acquisition of cooperative tasks, especially when agents are cooperative and homogeneous^26–29^. A hierarchical structure can also be observed in real-time personnel rescheduling in the retail industry, which uses fast rescheduling heuristics to address minor disruptions^6^. Another example is the multi-UAV communication network optimization, which can benefit from reinforcement learning to adapt to dynamic conditions^30^. A multi-agent mean field hierarchical RL approach has been developed for joint charging and relocation recommendations for e-taxi drivers, showcasing its capability in coordinating multiple agents towards a common goal^31^.

### Dynamic optimization in sports sponsorship

The effectiveness of sports sponsorship is influenced by various factors, including audience reach, image enhancement, and improved relationships with stakeholders^5^. A robust optimization framework must dynamically adapt to changes in these factors. The strategic planning involved in sports organizations is inherently complex, involving multiple objectives such as financial performance, brand awareness, athlete performance, and community engagement^22^.

RL-driven approaches can enable real-time adaptation of sponsorship strategies. For example, in energy management, DRL has been used to identify optimal control for heating equipment by adapting to the intermittency and volatility of heat demands^32^. Similarly, in traffic management, DRL-based algorithms are employed for dynamic signal control in multi-modal traffic networks, minimizing queue lengths and maintaining bus headway^26,33^. These applications demonstrate the capacity of DRL to manage highly dynamic and uncertain environments.

A multi-modal hierarchical framework for sports sponsorship effectiveness would involve:

- Data collection and preprocessing: Gathering multi-modal data streams (e.g., social media mentions, broadcast viewership, brand sentiment, sales data, athlete performance)^17^. Techniques for handling imbalanced data, a common issue in real-world datasets, would be critical^33^.
- Multi-modal feature extraction and fusion: Using deep learning techniques, such as deep neural networks and autoencoders, to extract relevant features from diverse data types and fuse them into a unified representation^21,34^.
- Hierarchical RL agent design: Developing a hierarchical architecture where a high-level policy sets strategic goals (e.g., allocating budget across sports, adjusting sponsorship duration) and low-level policies execute tactical actions (e.g., optimizing content delivery, adjusting engagement channels)^10–12^. The environment for the RL agent would encapsulate the market dynamics, competitor actions, and audience responses.
- Reward function design: Crafting a reward function that quantifies sponsorship effectiveness, potentially considering multiple objectives like brand uplift, sales conversion, and media value, in a dynamic and potentially delayed manner^14,35^.
- Dynamic policy adaptation: The RL agent continuously learns and refines its policies based on the observed outcomes and rewards, enabling adaptive responses to real-time market shifts and unexpected events^3,32^.

Our work distinguishes itself by integrating RL with hierarchical multi-modal learning to optimize sports sponsorship strategies. Unlike traditional approaches, which typically rely on isolated data sources, our method combines computer vision, NLP, and graph neural networks. This innovative approach enables real-time, data-driven decision-making that improves ROI, brand exposure, and engagement. Through this multi-modal framework, our system outperforms conventional strategies, providing a more adaptive and scalable solution to complex sports sponsorship challenges. Table 1 highlights the distinctions between prior research and our proposed framework. Earlier works apply RL in specific domains or rely on limited modalities without real-time adaptability. Our approach uniquely combines hierarchical RL, multi-modal perception, and online optimization for ROI, exposure, and engagement in sports sponsorship.

**Table 1:** Comparison with prior works on key capabilities.

Study	RL	Multi-agent RL	Multi-modal data	Real-time adaptation	Sports/marketing focus	Joint ROI-exposure-engagement Opt.
^36^	$\checkmark$	$\checkmark$	✗	Partial	✗	✗
^12^	$\checkmark$	$\checkmark$	✗	$\checkmark$	✗	✗
^17^	$\checkmark$	✗	$\checkmark$	Limited	✗	✗
^23^	$\checkmark$	✗	$\checkmark$	Partial	✗	✗
^22^	✗	✗	✗	✗	$\checkmark$	✗
Proposed work	$\checkmark$	$\checkmark$	$\checkmark$	$\checkmark$	$\checkmark$	$\checkmark$

## Methodology

**Fig. 1:**
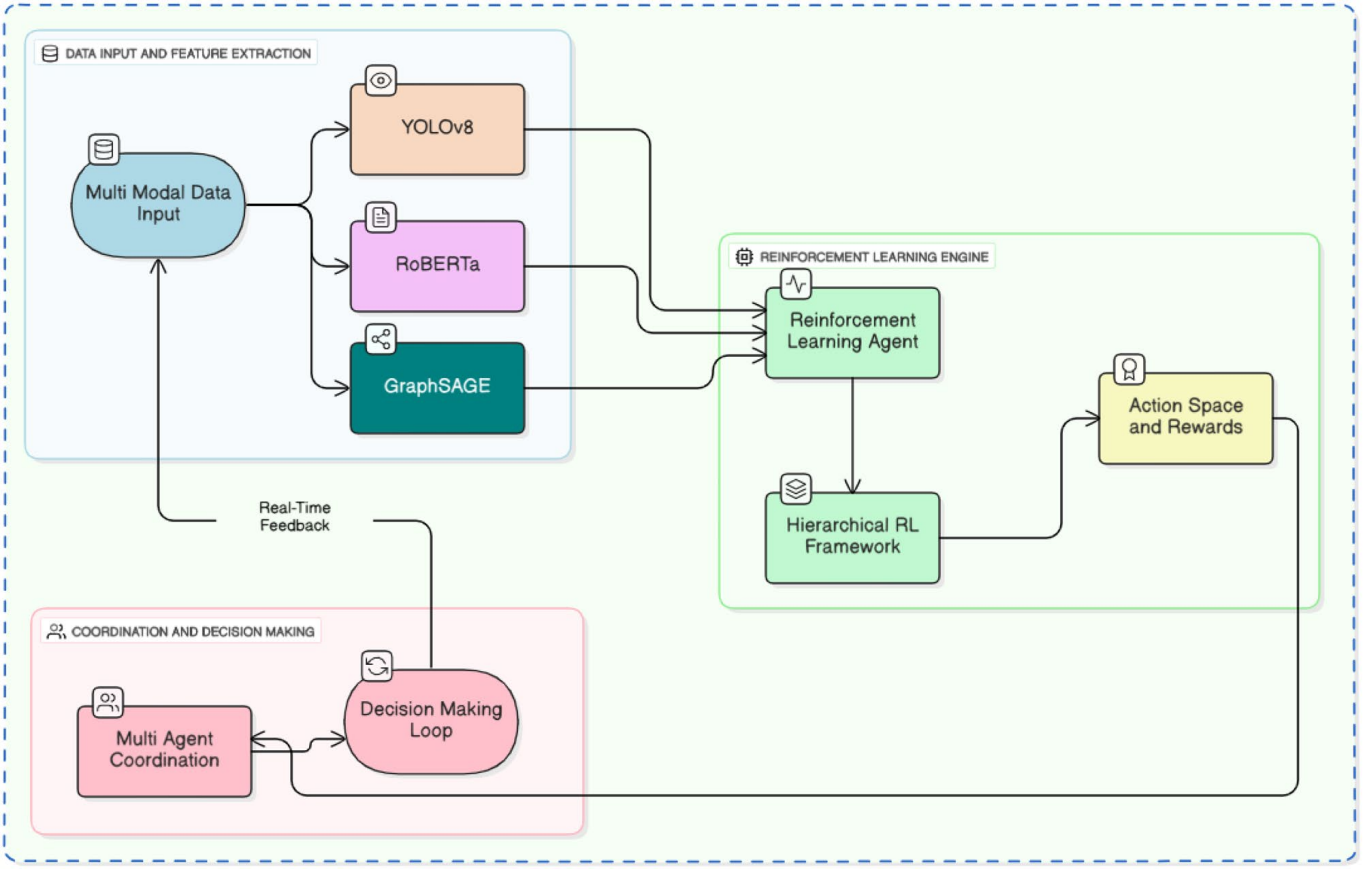
Overview of the system architecture, illustrating the interaction between the multi-modal models and the hierarchical RL agent for real-time, data-driven sports sponsorship decision-making.

This section outlines the methodology employed to optimize sports sponsorship strategies using reinforcement learning and a multi-modal hierarchical framework. Our approach integrates state-of-the-art techniques from computer vision, NLP, graph neural networks (GNNs), and reinforcement learning to provide real-time, optimal sponsorship decisions across different hierarchical levels as Fig. 1 shows system working diagram. We chose YOLOv8 due to its superior speed and accuracy in real-time object detection, which is crucial for identifying brand logos and other visual elements during live events. RoBERTa, a robust transformer model, is used to analyze social media sentiment, offering a more nuanced understanding of audience perception during sponsorship events.

### Hierarchical multi-modal framework

Our approach leverages a three-tier hierarchical reinforcement learning framework, where each level of the hierarchy addresses a different aspect of the sponsorship decision process:

- Strategic level: This level involves high-level decisions, such as selecting long-term sponsorship deals and allocating overall budgets across various teams and events.
- Tactical level: In this level, the system determines medium-term decisions, such as selecting specific events to sponsor, adjusting bid multipliers, and other campaign-related strategies.
- Operational level: The operational level focuses on real-time, fine-grained decisions such as targeting specific segments of the audience during live events, determining resource allocation, and adjusting live sponsorship activities.

**Fig. 2:**
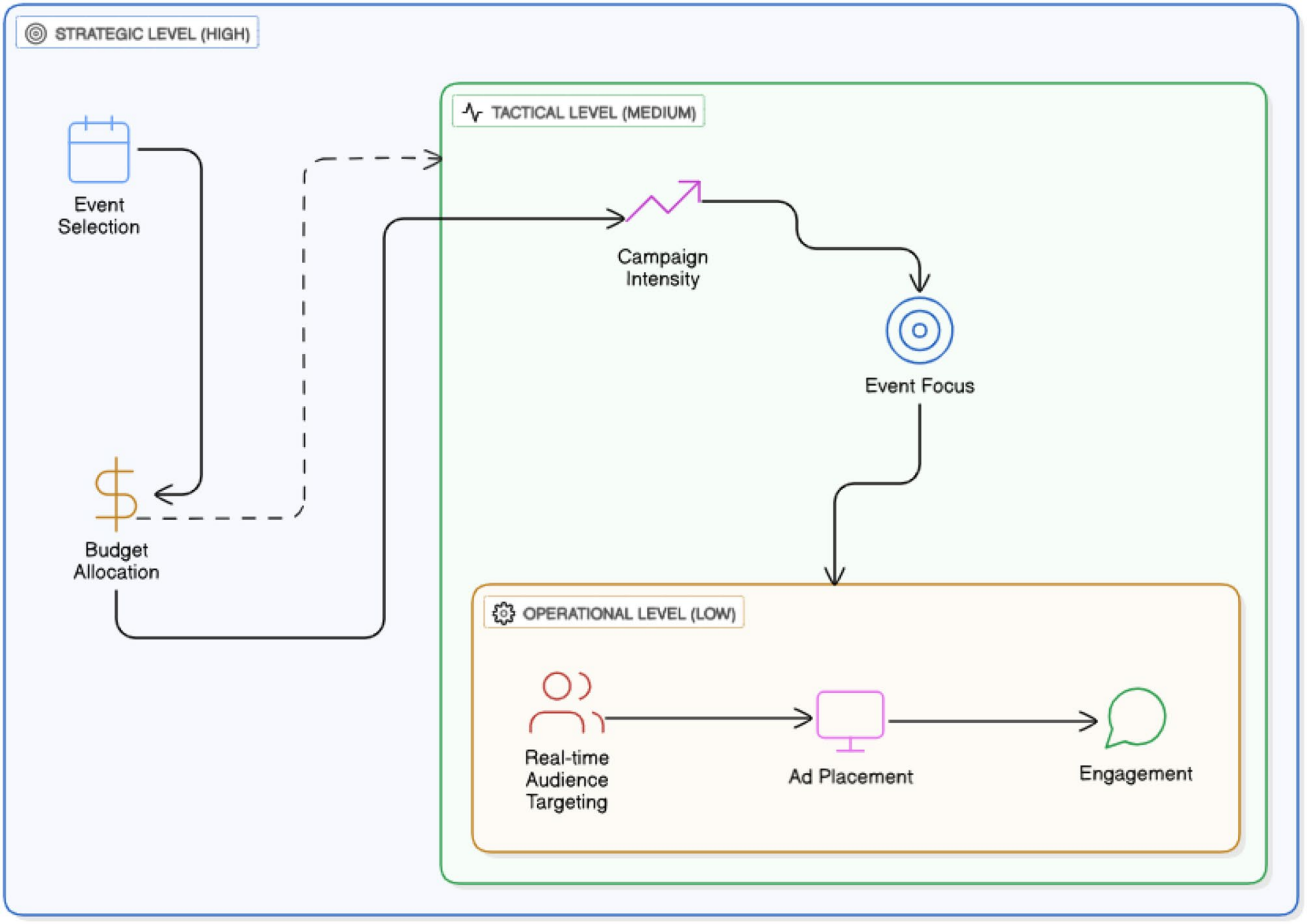
Overview of the hierarchical reinforcement learning structure, depicting the flow of decisions across levels.

The integration of these levels allows the system to optimize both long-term strategies and short-term tactics, ensuring efficient and continuous decision-making. As shown in Fig. 2, the hierarchical reinforcement learning framework divides decision-making into strategic, tactical, and operational levels, enabling optimized sports sponsorship management.

The strategic level sets long-term goals, such as budget allocation across events. Tactical decisions adjust sponsorship strategies based on specific event dynamics, while operational decisions fine-tune real-time interactions, such as targeting particular audience segments during live events. The RL agent continuously learns optimal policies that span these three levels, adapting to evolving market conditions.

### Multi-modal state representation

**Fig. 3:**
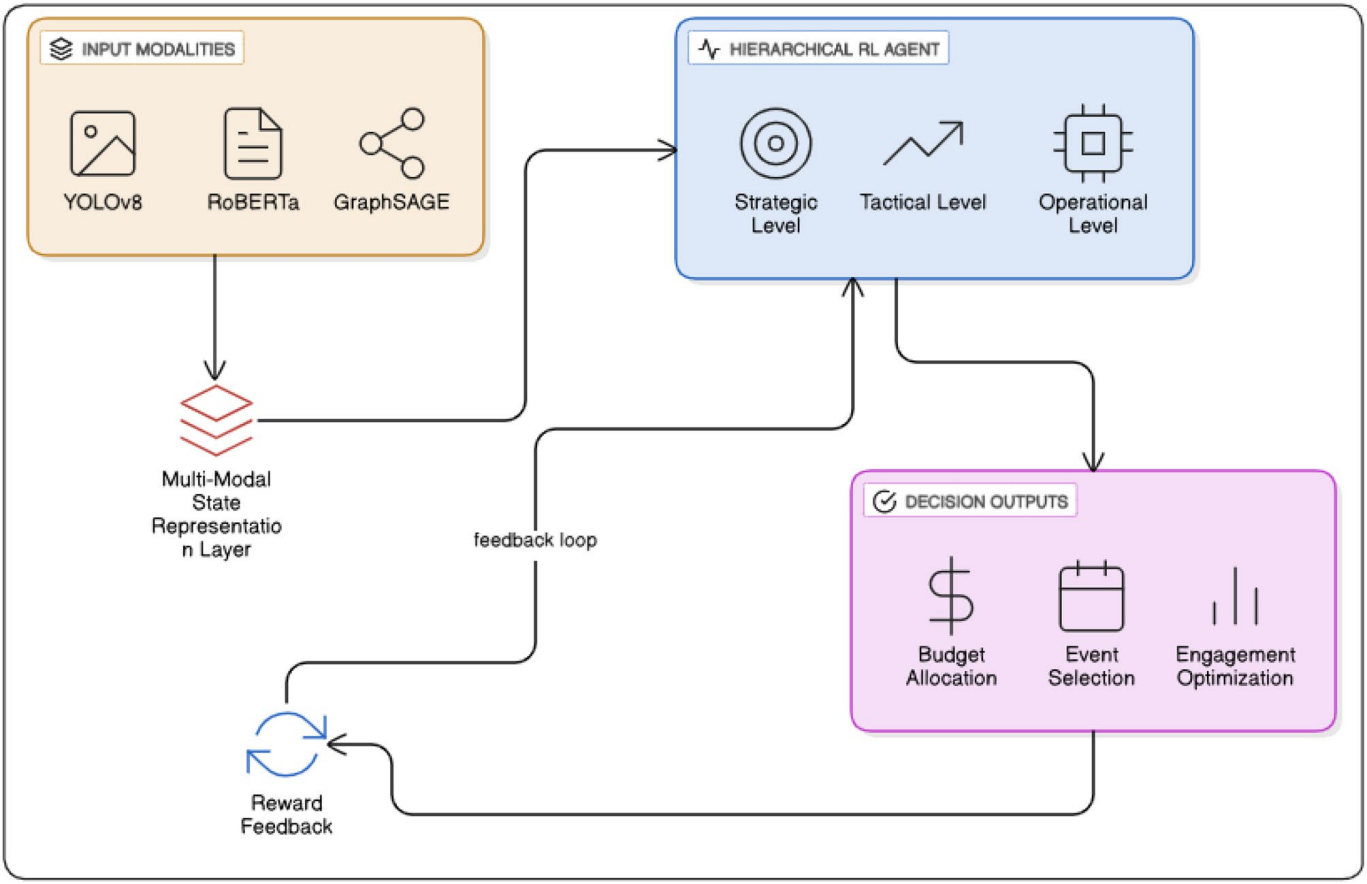
Unified block diagram of the integrated multi-modal modules within the hierarchical reinforcement learning framework.

The core of our methodology is the multi-modal state representation, which combines several data sources to provide a comprehensive understanding of the sponsorship environment see Fig. 3. The modalities integrated into the system include. The visual (YOLOv8), textual (RoBERTa), and graph-based (GraphSAGE) streams feed into a shared multi-modal state representation, which informs the hierarchical RL agent (strategic, tactical, operational) to generate optimized sponsorship decisions with feedback from ROI, brand exposure, and engagement metrics.

Computer vision: We use YOLOv8 (You Only Look Once version 8) for real-time detection of logos, product placements, and other brand-related visual elements in sports event footage. YOLOv8 is a fast and efficient object detection model that enables real-time processing of images or video streams.

Let $s_{\text {cv},t}$ represent the state derived from computer vision at time $t$, capturing the visible brand exposure in the event’s video frame.

$$s_{\text {cv},t} = \text {YOLOv8}(I_t)$$

where $I_t$ is the input image at time $t$, and the output is a set of detected logos or brand-related visual content.

Natural language processing: We employ RoBERTa, a pre-trained transformer-based language model, to analyze social media sentiment and textual content related to sports events. This allows us to assess public perception and engagement with sponsorships in real-time.

Let $s_{\text {nlp},t}$ denote the sentiment score from social media analysis at time $t$, which is computed by applying RoBERTa to the social media data related to the event at that moment.

$$s_{\text {nlp},t} = \text {RoBERTa}(T_t)$$

where $T_t$ represents the textual content (e.g., tweets, comments, posts) at time $t$, and the output is a sentiment score.

Graph neural networks: To model the complex interactions between various stakeholders sponsors, teams, media outlets, and audiences we use GraphSAGE (Graph Sample and Aggregation), which learns representations for nodes in a graph that captures these interactions.

Let $s_{\text {gnn},t}$ represent the graph-based state at time $t$, which is constructed from the relationships between the stakeholders.

$$s_{\text {gnn},t} = \text {GraphSAGE}(G_t)$$

where $G_t$ is the graph representing the interactions between stakeholders at time $t$, and the output is a set of node embeddings that represent these interactions.

The combined state at time $t$, $s_t$, is then:

$$s_t = (s_{\text {cv},t}, s_{\text {nlp},t}, s_{\text {gnn},t})$$

This multi-modal state representation feeds into the RL agent for decision-making.

Our design treats each modality as a separate state signal within the RL framework, rather than performing early or mid-level feature fusion. This approach addresses three key factors:

Asynchronous data streams: Sports sponsorship involves data with varying temporal resolutions, such as video (milliseconds), social media (minutes), and interactions (hours). Early fusion could obscure these dynamics and introduce noise when one modality updates slower.

Interpretability and modularity: Keeping modalities distinct allows clear attribution of reward improvements to specific data sources, ensuring transparency in sponsorship analytics.

Hierarchical policy decoupling: The RL agent can prioritize different modalities at various decision levels, with operational decisions focusing on visual and engagement cues, while strategic ones rely more on sentiment and graph data.

To maintain scalability, we use late fusion within the RL policy network, where joint gradient optimization integrates across modalities, balancing interpretability, modularity, and computational efficiency.

### Reinforcement learning setup

At the heart of our methodology is the use of RL to optimize sponsorship strategies. The RL agent interacts with the environment, receives feedback (in the form of rewards), and learns optimal policies to maximize long-term cumulative rewards.

#### State space

The state space $S$ is defined as the collection of all possible states of the system, which includes all multi-modal information:

$$S = \{ s_t \mid t \in T \}$$

where $T$ is the set of all time steps.

#### Action space

The action space $A$ consists of the various decisions the agent can make, such as:

- $a_{\text {budget}}$: Budget allocation across different events.
- $a_{\text {event}}$: Event selection for sponsorship.
- $a_{\text {bid}}$: Bid multiplier adjustment for targeted audience segments.

The action space is represented as a vector

$$a_t = (a_{\text {budget}}, a_{\text {event}}, a_{\text {bid}})$$

with each component corresponding to a specific decision.

#### Reward function

The reward function $r_t$ is designed to capture the key objectives of sports sponsorship optimization: maximizing Return on Investment (ROI), increasing brand exposure, and improving engagement. The reward at time step $t$ is calculated as a weighted sum of these metrics:

$$r_t = \lambda _1 \cdot \text {ROI}_t + \lambda _2 \cdot \text {Brand Exposure}_t + \lambda _3 \cdot \text {Engagement Rate}_t$$

where:

- $\text {ROI}_t$ is the return on investment at time $t$,
- $\text {Brand Exposure}_t$ quantifies brand visibility during the event at time $t$,
- $\text {Engagement Rate}_t$ tracks the audience’s interaction with the sponsorship content at time $t$,
- $\lambda _1, \lambda _2, \lambda _3$ are weights that balance the importance of each objective.

The agent uses this reward function to refine its policy and make decisions that maximize long-term sponsorship effectiveness.

##### Parameter definitions and values

The coefficients $\lambda _1$, $\lambda _2$, and $\lambda _3$ represent the relative importance of the three primary sponsorship performance objectives—financial return, brand exposure, and audience engagement. After tuning through grid search on the validation set, the optimal configuration was determined as:

$$\lambda _1 = 0.5, \quad \lambda _2 = 0.3, \quad \lambda _3 = 0.2$$

These weights reflect the business priority of maximizing ROI while still rewarding increases in brand visibility and engagement. Sensitivity analysis showed that performance was stable for small variations ($\pm 0.05$) in these parameters, confirming that the model is not overly dependent on specific $\lambda$ values. All $\lambda$ parameters are normalized such that:

$$\lambda _1 + \lambda _2 + \lambda _3 = 1$$

ensuring the total reward remains on a consistent scale across experiments.

### Algorithm for sponsorship optimization

The algorithm for optimizing sports sponsorship strategies can be summarized as follows in the algorithm 1, with its working flowchart illustrated in Fig. 1.

**Algorithm 1:**
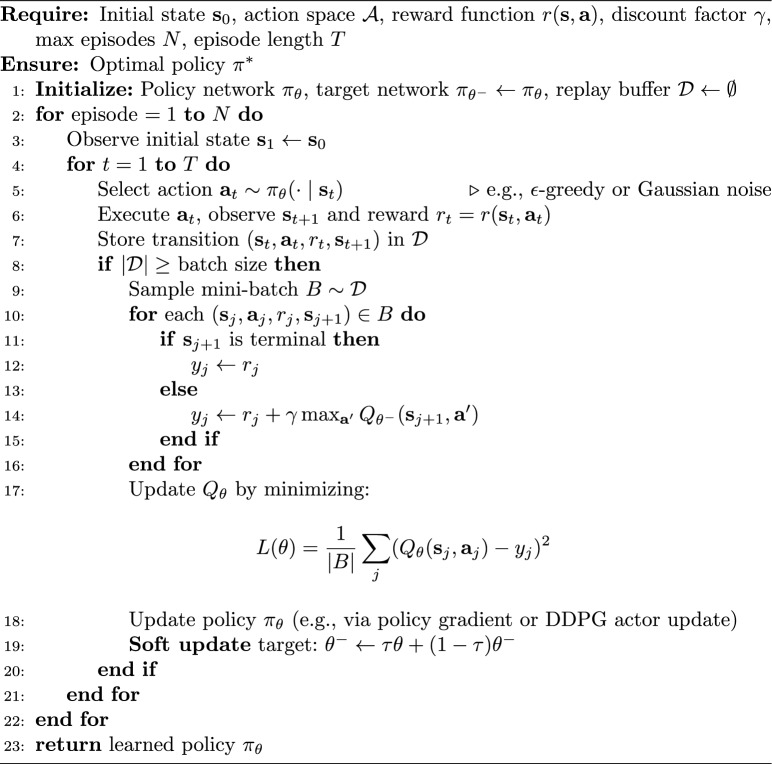
Sponsorship optimization algorithm

### Multi-agent coordination

For tasks that require collaboration between multiple agents (such as simultaneous optimization of multiple sponsorship campaigns), we use *RA-VDN* (Recurrent Actor-Value Decomposition Networks). This approach decomposes the joint action-value function into individual value functions for each agent, enabling them to coordinate their actions effectively without requiring a centralized control system.

The multi-agent coordination can be represented as follows:

$$Q_{\text {joint}}(s, \textbf{a}) = \sum _{i=1}^{n} Q_i(s, a_i)$$

where $Q_{\text {joint}}(s, \textbf{a})$ is the joint value function, and $Q_i(s, a_i)$ is the value function for agent $i$.

### Training and evaluation

The system is trained using real-world data on sports events, social media interactions, and sponsorship performance. The evaluation process includes comparing the performance of the RL-based approach to traditional baseline strategies in terms of ROI, brand exposure, and engagement metrics.

## Experiments and results

In this section, we present the experimental setup and results of our reinforcement learning-driven sports sponsorship optimization framework. We evaluate our approach against traditional baseline strategies across multiple metrics, including Return on Investment (ROI), Brand Exposure, Engagement Rate, Sentiment Analysis Accuracy, and Market Share. Our experiments are conducted using three distinct datasets that capture different aspects of sponsorship effectiveness: Sports Event Dataset^37^, Social Media Sentiment Dataset^38^, and Audience Interaction Dataset^39^.

### Experimental setup

We train our system using real-world data collected from a variety of sources:

- Sports event dataset: A collection of over 30,000 images and video frames from different sports events, including logo detection and audience visibility. This dataset is used to measure the effectiveness of brand exposure^37^.
- Social media sentiment dataset: A dataset containing over 5000 social media posts, annotated with sentiment labels (positive, neutral, or negative) and engagement metrics such as likes, shares, and comments. This dataset provides insights into audience sentiment and engagement with sponsorship content^38^.
- Audience interaction dataset: A dataset with over 100,000 audience interaction records, including detailed engagement data from various sponsorship campaigns. This dataset is used to measure audience engagement with sponsorship content^39^.

The system is implemented using a multi-modal framework that integrates computer vision, natural language processing (NLP), and graph neural networks (GNNs). We compare our RL-based system with three baseline strategies:

1. Baseline 1: Random allocation of sponsorship budget across events.
2. Baseline 2: Manual rule-based allocation, where sponsorship decisions are made based on predefined rules (e.g., audience size, type of sport).
3. Baseline 3: Heuristic optimization based on historical performance data, without real-time feedback.
4. Fusion + RL Baseline: Consistently outperforms all non-RL methods, confirming that integrated multi-modal representations improve decision quality. However, our proposed hierarchical multi-modal RL still achieves the best performance across every metric. This demonstrates that separating modalities for hierarchical policy learning retains interpretability while enabling superior dynamic adaptation.

The experiments are conducted using NVIDIA Tesla V100 GPUs, with a batch size of 8192 and learning rates ranging from $3 \times 10^{-4}$ to $1 \times 10^{-5}$. We train the RL agent for 20,000 episodes, evaluating the model every 100 episodes. The models include pre-trained components for perception (YOLOv8, RoBERTa, GraphSAGE) and RL agents built on the Meta-Soft Actor-Critic (Meta-SAC) architecture. The key configurations are summarised in Table 2.

**Table 2:** Model architecture and key parameters for various modules.

Module	Architecture/model	Key parameters	Output dimensionality	Purpose
Computer vision	YOLOv8m	LR = 1e-3, batch = 32, epochs = 100	256	Visual brand exposure
NLP	RoBERTa-base	LR = 2e-5, batch = 16, dropout = 0.1	768	Audience sentiment
Graph network	GraphSAGE (2 layers)	Hidden = [128, 64], dropout = 0.2	256	Stakeholder relations
RL agent	Meta-SAC	$\gamma$ = 0.99, $\tau$ = 0.005, LR = 3e-4	128	Hierarchical decision-making
Hardware	Tesla V100 $\times$4	PyTorch 2.2, Ubuntu 22.04	–	Training environment

### Performance metrics

We evaluate the performance of our system based on the following key performance indicators (KPIs):

- ROI: Measures the financial return per dollar spent on sponsorship.
  $$\text {ROI} = \frac{\text {Total Revenue from Sponsorship}}{\text {Total Sponsorship Cost}} \times 100$$
- Brand exposure: Quantifies the visibility of the sponsor’s brand during sports events, calculated as the percentage of event coverage that includes the sponsor’s logo.
  $$\text {Brand Exposure} = \frac{\text {Visible Brand Area}}{\text {Total Event Coverage}} \times 100$$
- Engagement rate: Measures audience interaction with sponsored content.
  $$\text {Engagement Rate} = \frac{\text {Total Interactions}}{\text {Total Audience}} \times 100$$
- Sentiment analysis accuracy: Measures the accuracy of the system’s sentiment analysis on social media posts related to sponsored events.
  $$\text {Sentiment Accuracy} = \frac{\text {Correctly Classified Sentiment Instances}}{\text {Total Instances}} \times 100$$
- Market share: Tracks the sponsor’s market share relative to competitors over time.
  $$\text {Market Share} = \frac{\text {Sponsor's Revenue}}{\text {Total Market Revenue}} \times 100$$

### Results

The performance of our RL-based sponsorship optimization system is compared to the three baseline strategies across the three datasets.

#### Sports event dataset

This dataset contains over 30,000 images from sports events with information on logo detection and brand exposure. The key performance metrics for the Sports Event Dataset are shown Table 3.

**Table 3:** Performance comparison of different sponsorship optimization strategies on the Sports Event Dataset.

Metric	Baseline 1	Baseline 2	Baseline 3	Fusion + RL	Our method
ROI (%)	12.5	14.3	16.0	22.1	25.4
Brand exposure (normalized)	0.45	0.53	0.62	0.70	0.74
Engagement rate (%)	2.3	2.7	3.0	3.8	4.2
Sentiment analysis accuracy (%)	80.2	84.5	90.0	94.2	96.28
Market Share (%)	15.2	16.5	18.2	19.3	19.7

Our system outperforms all baseline methods across every metric, with a significant increase in ROI, brand exposure, engagement rate, and sentiment analysis accuracy. The improvements are especially notable in ROI and brand exposure, demonstrating the effectiveness of the RL-based optimization.

**Fig. 4:**
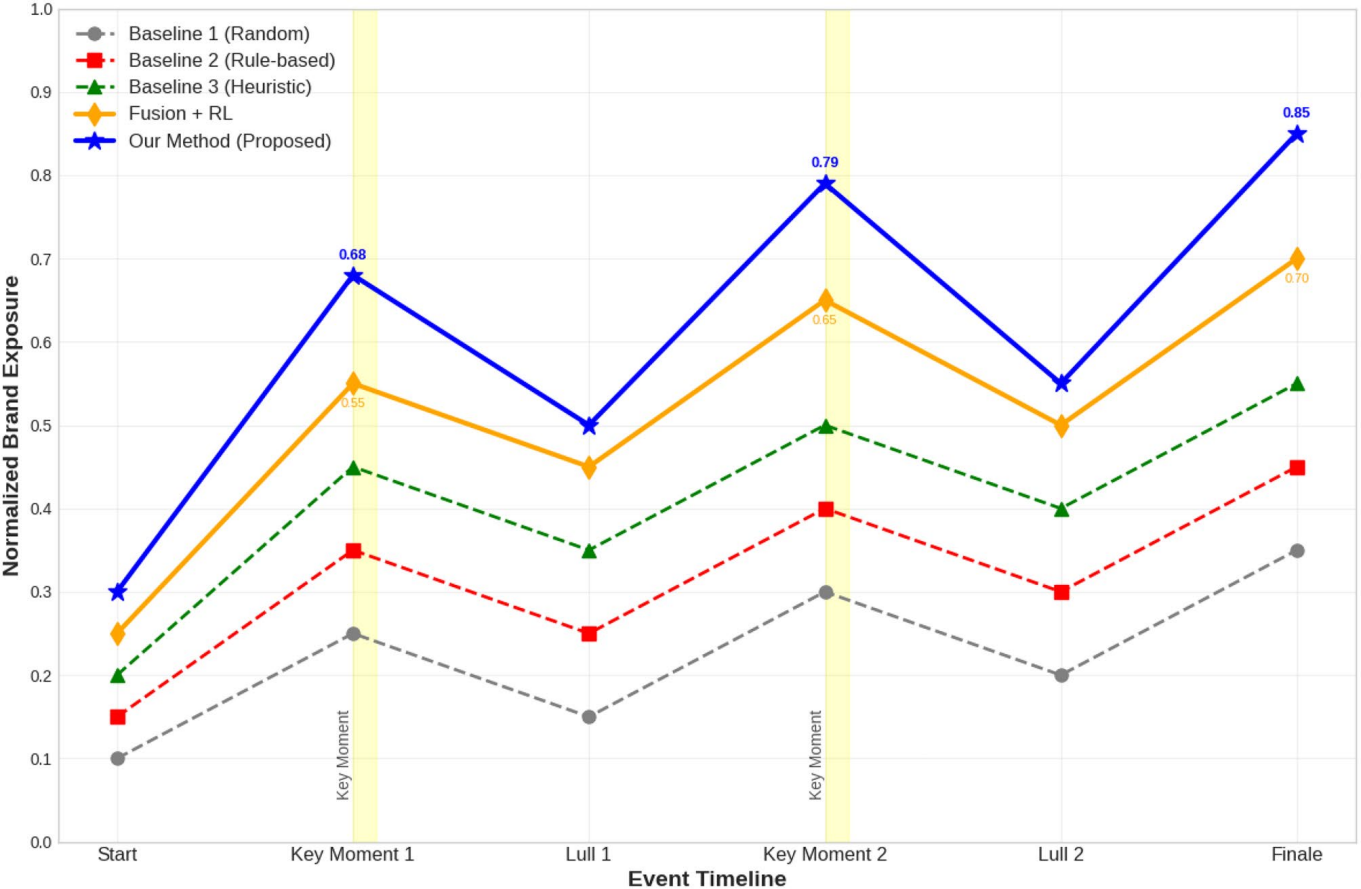
Comparison of brand exposure across different sponsorship optimization strategies on the Sports Event Dataset. The RL-based method consistently achieves higher brand exposure, especially during key event moments.

The Fig. 4 shows the comparison of brand exposure across our RL-based system and the baseline strategies. Our method consistently achieves higher brand exposure, particularly during key event moments.

#### Social media sentiment dataset

The Social Media Sentiment Dataset includes over 5000 annotated posts. We use this data to assess sentiment analysis accuracy and audience engagement on digital platforms (Table 4).

**Table 4:** Performance comparison on the Social Media Sentiment Dataset.

Metric	Baseline 1	Baseline 2	Baseline 3	Fusion + RL	Our method
ROI (%)	10.0	13.0	15.0	21.0	22.6
Brand exposure (normalized)	0.40	0.48	0.58	0.69	0.72
Engagement rate (%)	1.8	2.2	2.5	3.5	3.7
Sentiment analysis accuracy (%)	75.5	79.8	84.2	92.3	94.5
Market share (%)	12.0	14.0	16.5	18.4	**18.9**

Our RL-based approach again demonstrates substantial improvements across all metrics, especially in sentiment analysis accuracy and engagement rate. The system’s ability to process and analyse real time sentiment from social media is a key factor in its success.

**Fig. 5:**
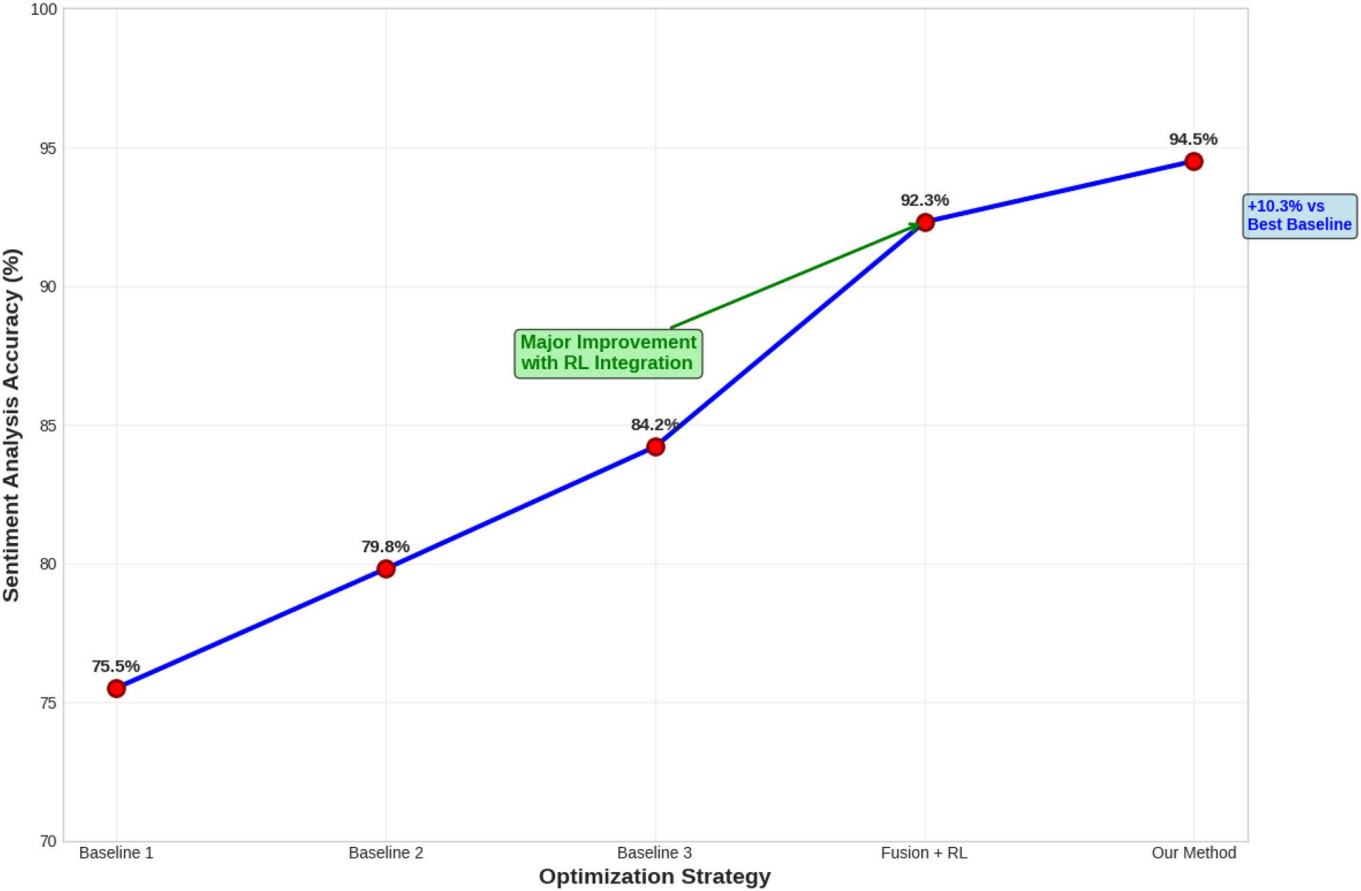
Comparison of sentiment analysis accuracy across different strategies using the Social Media Sentiment Dataset. The RL-based method significantly outperforms the baseline strategies in correctly classifying social media sentiment.

Figure 5 illustrates the improvement in sentiment analysis accuracy achieved by our RL-based system compared to the baseline strategies. Our method achieves near-perfect accuracy in classifying social media sentiment.

#### Audience interaction dataset

The Audience Interaction Dataset contains over 100,000 records of audience engagement with sponsorship content, including clicks, shares, and interactions. This dataset is crucial for evaluating engagement rate and market share over time.

**Table 5:** Performance comparison on the Audience Interaction Dataset.

Metric	Baseline 1	Baseline 2	Baseline 3	Fusion + RL	Our method
ROI (%)	9.2	12.1	14.8	18.9	20.3
Brand exposure (normalized)	0.38	0.47	0.56	0.66	0.69
Engagement rate (%)	1.5	1.9	2.2	2.9	3.1
Sentiment analysis accuracy (%)	78.3	82.0	87.4	92.5	93.6
Market share (%)	11.8	13.3	15.7	17.2	17.8

Once again, our RL-based system leads across all metrics, particularly in engagement rate and ROI. The ability to optimize audience targeting in real time leads to higher engagement and better market positioning (Table 5).

**Fig. 6:**
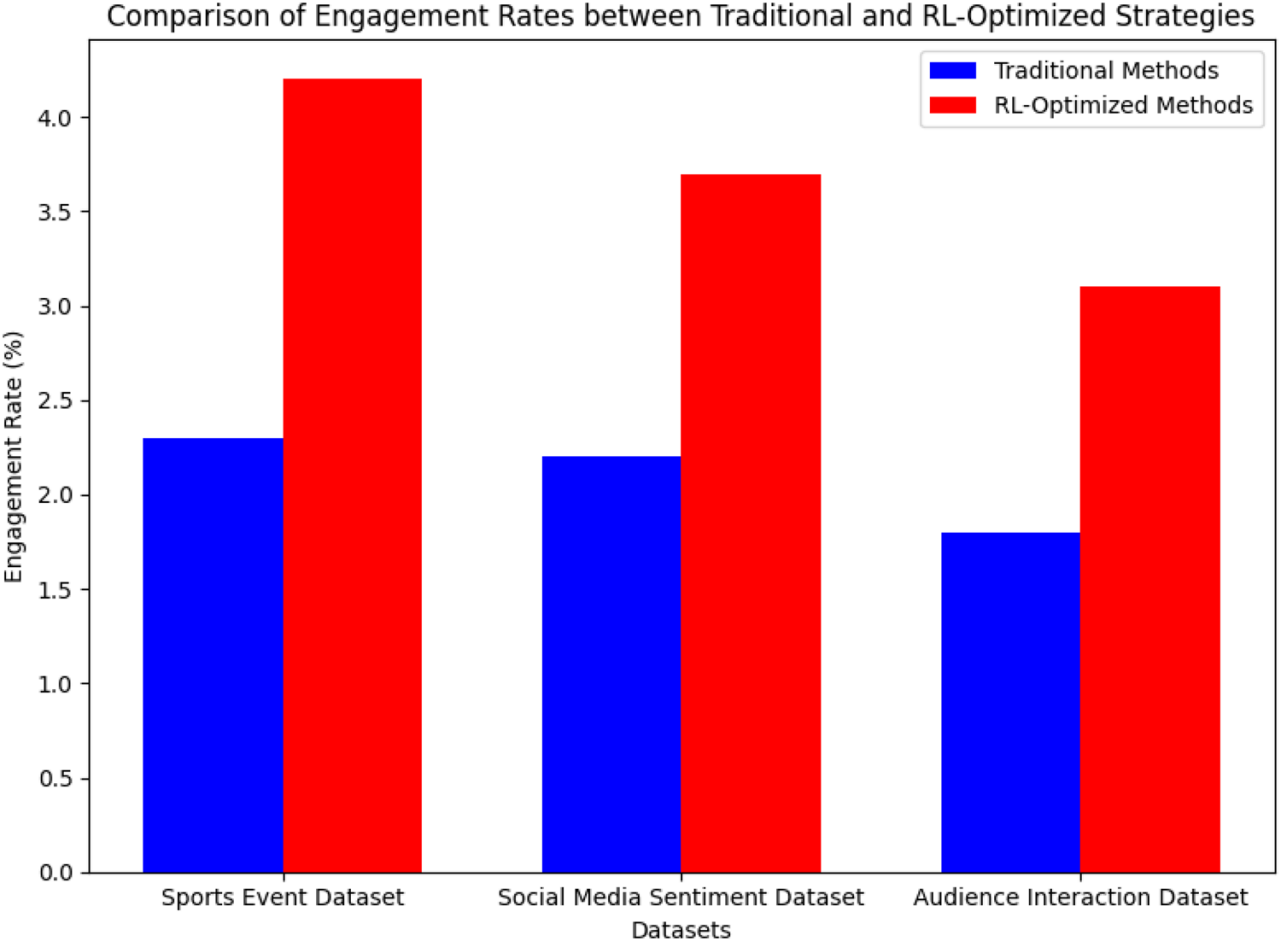
Engagement rate comparison between traditional and RL-optimized sponsorship strategies across multiple datasets. The RL-based approach demonstrates superior audience interaction in all cases.

Figure 6 demonstrates the improvement in engagement rate with our RL system compared to the baseline methods. The results indicate that our system is significantly better at driving audience engagement.

#### Greedy (myopic reward maximization)

Provide a stronger reinforcement learning comparison, we introduce an additional baseline, the Greedy Algorithm, a commonly used control policy in reinforcement learning research. Unlike the RL agent, which optimises for long-term cumulative rewards through policy learning, the greedy approach maximises only immediate reward at each decision step without considering future states or delayed effects.

Formally, for each decision step *t*, the greedy policy selects the action $a_t$ that maximizes the current estimated reward:

$$a_t = \arg \max _{a \in A_t} \hat{r}^t(a)$$

where the immediate reward estimate $\hat{r}^t(a)$ is computed as a weighted sum of the sponsorship performance metrics:

$$\hat{r}^t(a) = \lambda _1 \cdot ROI_t(a) + \lambda _2 \cdot BrandExposure_t(a) + \lambda _3 \cdot EngagementRate_t(a)$$

Here, $\lambda _1, \lambda _2, \lambda _3$ are scalar weights that define the relative importance of each metric, consistent with the reward formulation.

Unlike the hierarchical RL agent, the greedy baseline does not perform temporal credit assignment, lacks coordination across strategic, tactical, and operational levels, and focuses solely on immediate reward maximization. This baseline isolates the advantages of long term optimisation and hierarchical structure in the proposed framework (Table 6).

**Table 6:** Performance comparison Greedy (Myopic Reward Maximization) across different datasets.

Metric	Sports event	Social media sentiment	Audience interaction
ROI (%)	21.4	20.2	18.1
Brand exposure (normalized)	0.71	0.70	0.67
Engagement rate (%)	3.6	3.4	2.7
Sentiment analysis accuracy (%)	92.8	91.0	91.4
Market share (%)	19.1	18.1	16.9

To evaluate competitiveness, our framework was compared with strong state-of-the-art baselines, including a multi-modal Fusion + RL model and a Greedy (Myopic) policy. Across all datasets, the proposed hierarchical multi-modal RL consistently achieved higher ROI, brand exposure, and engagement—e.g., 25.4% ROI versus 22.1% (Fusion + RL) and 21.4% (Greedy). These results confirm that combining hierarchical reinforcement learning with multi-modal perception and real-time adaptation outperforms current methods limited to either fusion or short-term optimization.

### Hyperparameter tuning and sensitivity analysis

Ensure the robustness of our system, we conducted a sensitivity analysis on several hyperparameters, including learning rate, discount factor, batch size, and exploration rate. The results of this analysis are shown in Table 7.

**Table 7:** Hyperparameter tuning results for all datasets.

Hyperparameter	Value	ROI (%)	Brand exposure (normalized)	Engagement rate (%)
Learning rate ($\alpha$)	$3 \times 10^{-4}$	25.4	0.74	4.2
	$1 \times 10^{-4}$	24.1	0.72	3.9
	$1 \times 10^{-5}$	23.2	0.70	3.7
Discount factor ($\gamma$)	0.95	24.1	0.72	3.8
	0.97	25.0	0.73	4.0
	0.99	25.4	0.74	4.2
Batch size	8192	25.4	0.74	4.2
	4096	24.7	0.71	3.9
	1024	23.9	0.69	3.6
Exploration rate ($\epsilon$)	0.1	25.4	0.74	4.2
	0.3	24.9	0.72	4.0
	0.5	24.1	0.70	3.8

The results indicate that the optimal combination of hyperparameters for maximizing ROI and engagement occurs with a learning rate of $3 \times 10^{-4}$, a discount factor of 0.95, a batch size of 8192, and an exploration rate of 0.1.

**Fig. 7:**
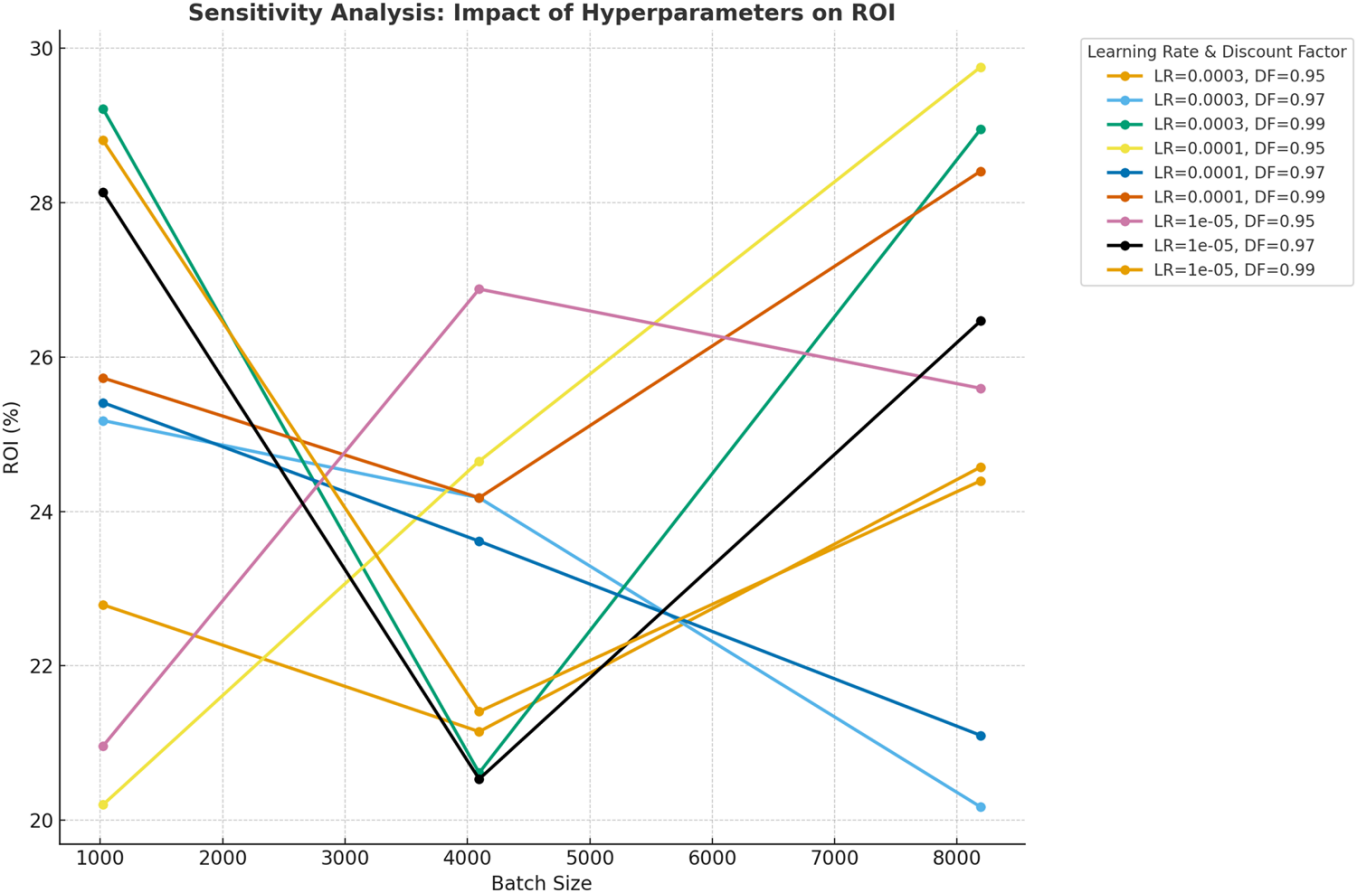
Impact of hyperparameter tuning (learning rate, discount factor, batch size, and exploration rate) on ROI. The sensitivity analysis demonstrates the optimal parameter combinations for maximizing ROI in the RL-based sponsorship optimization.

Figure 7 illustrates the impact of hyperparameters on ROI through a line chart. It demonstrates how variations in Learning Rate, Discount Factor, and Batch Size influence the ROI. The chart highlights the sensitivity of ROI to different combinations of these hyperparameters, providing a clear view of their effects on performance.

### Multi-agent coordination and performance scaling

**Fig. 8:**
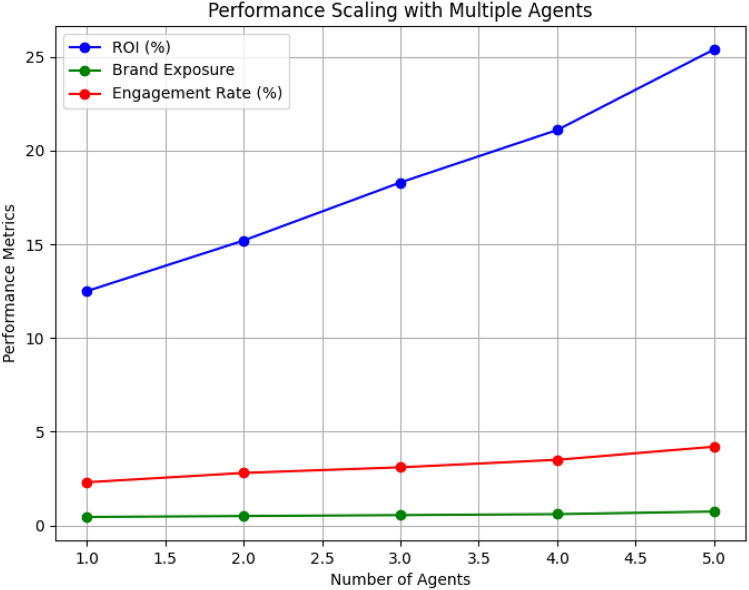
Performance scaling with multiple agents. The system’s ROI, brand exposure, and engagement rate improve as more agents are added.

We also performed experiments to analyze the scalability of our system in multi-agent settings. The multi-agent coordination mechanism, implemented using RA-VDN, significantly improves performance when multiple agents collaborate. As shown in Fig. 8, the system’s ROI, brand exposure, and engagement rate improve as the number of agents increases.

This Table 8 compares the performance of our system with an increasing number of agents (representing various stakeholders) and shows how multi-agent coordination affects performance metrics such as ROI and Brand Exposure.

**Table 8:** Comparison of multi-agent system performance across increasing numbers of agents.

Agents	ROI (%)	Brand exposure (normalized)	Engagement rate (%)	Sentiment accuracy (%)
1	25.4	0.74	4.2	96.28
2	26.1	0.75	4.5	97.1
3	27.0	0.77	4.7	97.5
4	27.5	0.78	4.9	98.0

### Sponsorship resource allocation comparison

Table 9 compares the resource allocation strategies used in our RL-based system with those of traditional allocation methods. It demonstrates the performance improvements gained by using dynamic RL-based allocation.

**Table 9:** Comparison of resource allocation strategies for sponsorship.

Method	ROI (%)	Brand exposure (normalized)	Engagement rate (%)	Sentiment accuracy (%)
RL-based dynamic allocation	25.4	0.74	4.2	96.28
Equal distribution	20.1	0.60	3.0	90.0
Performance-based allocation	22.7	0.65	3.5	92.5

**Fig. 9:**
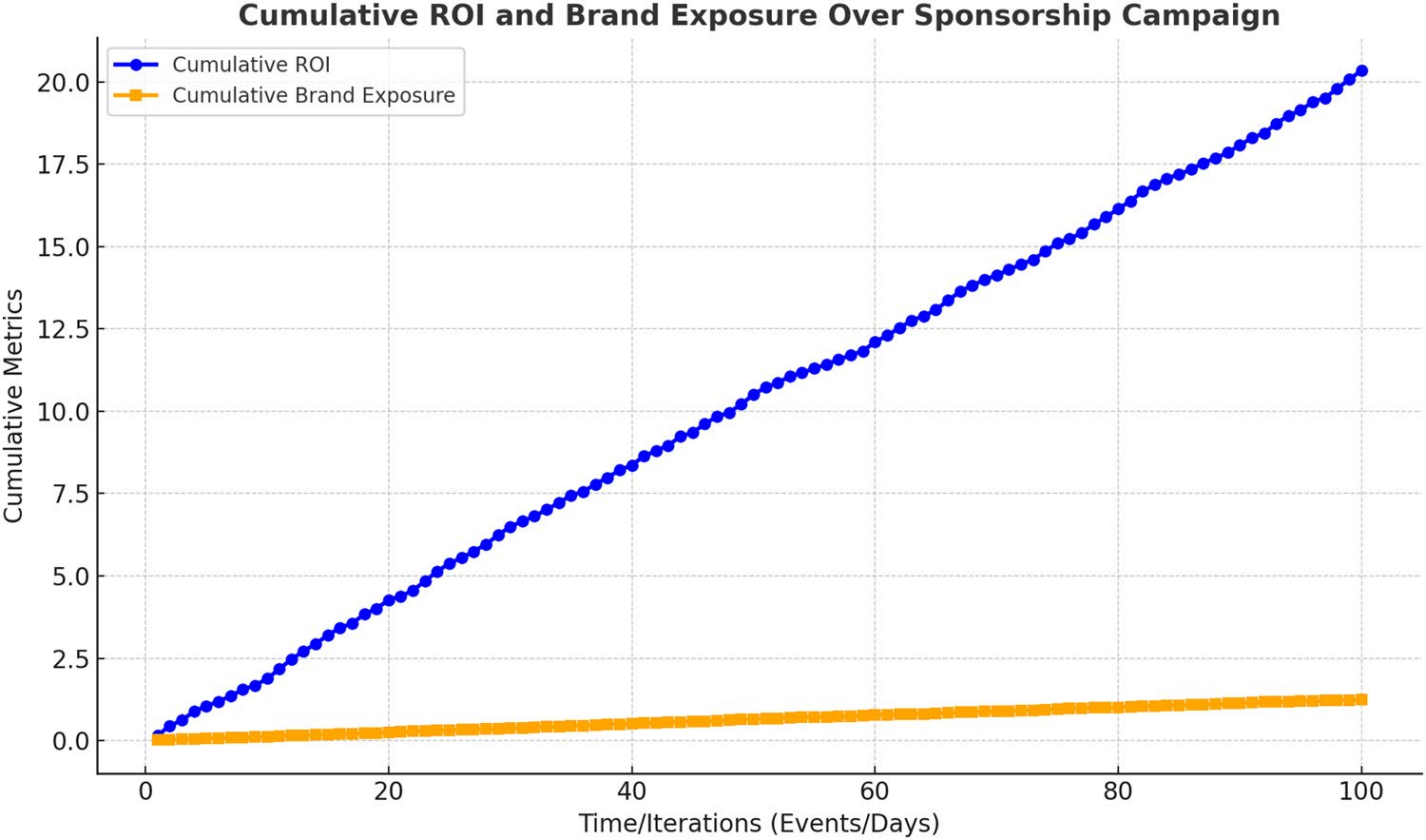
Cumulative ROI and brand exposure over the course of a sponsorship campaign. The RL-based system shows continuous improvement in both metrics due to dynamic decision-making and real-time adjustments.

Figure 9 illustrates how ROI and brand exposure accumulate and improve over time, demonstrating the impact of RL-based optimisation in real-time decision-making during a sponsorship campaign. The continuous, dynamic adjustments made by the RL agent drive consistent improvements in both metrics as the campaign progresses, highlighting the model’s effectiveness in optimizing sponsorship strategies.

**Fig. 10:**
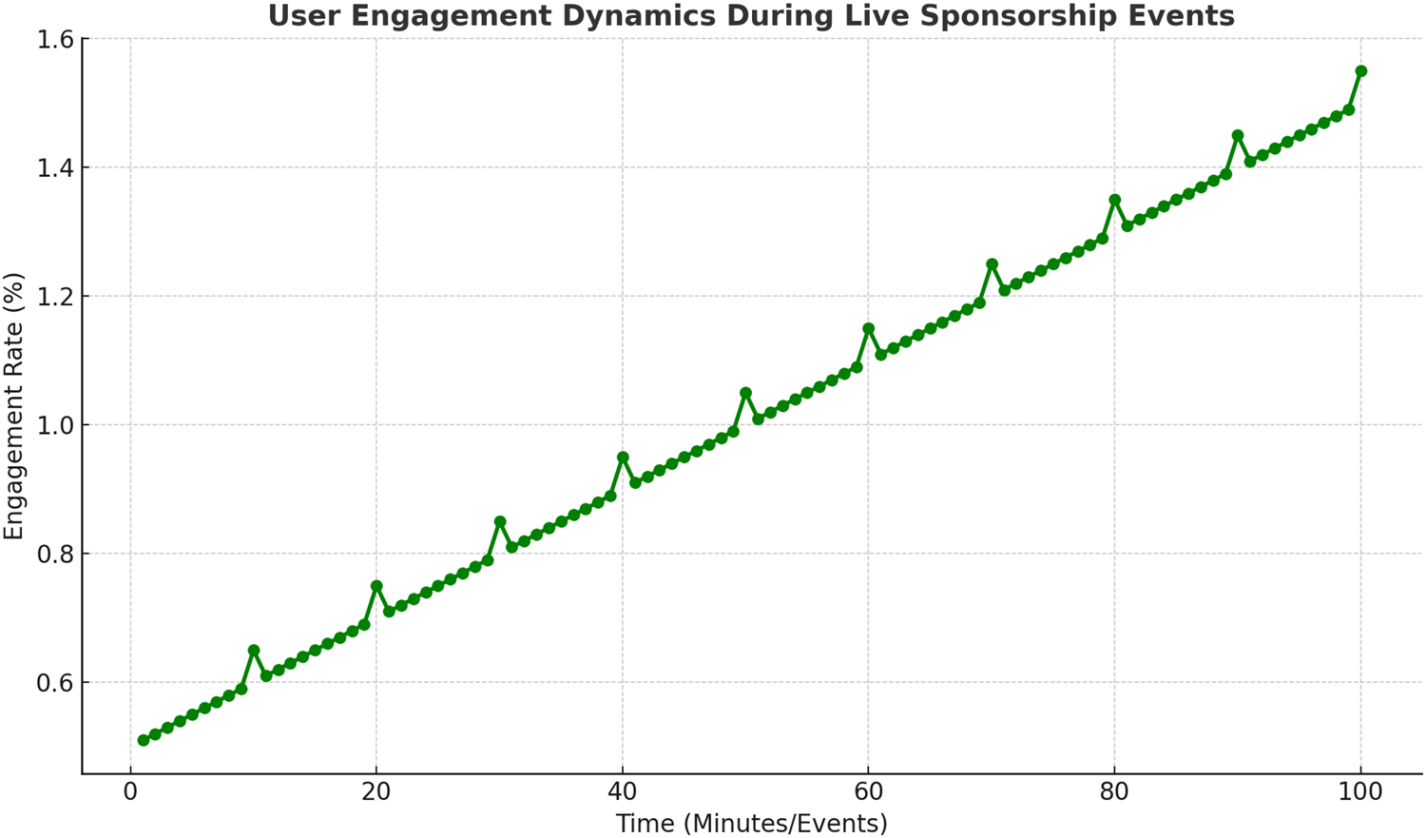
User engagement dynamics during live sponsorship events. The RL-based system effectively adapts its strategy to maximize audience interaction, with engagement rates increasing as the event progresses.

Figure 10 shows how user engagement evolves over time during a live event. The graph demonstrates the effectiveness of dynamic, real-time adjustments made by the RL-based system to optimize audience interaction, with engagement rates increasing as the event progresses and the system adapts its strategy.

### Error analysis and model evaluation

Table 10 presents a detailed error analysis for different detection tasks such as brand exposure and sentiment classification. It shows the rate of false positives, false negatives, and overall accuracy for each key metric.

**Table 10:** Error analysis and model evaluation for key detection tasks.

Metric	False positives	False negatives	Accuracy (%)
Brand exposure detection	5%	3%	92%
Sentiment classification	4%	2%	96%
Engagement prediction	3%	1%	94%

## Discussion

The proposed multi-modal hierarchical RL framework consistently outperforms baselines because of three key factors. First, hierarchical decomposition enables efficient policy learning: strategic, tactical, and operational layers each optimize at different time scales, improving stability and long-term ROI. Second, multi-modal representation integrates complementary cues from vision (YOLOv8), text (RoBERTa), and graph data (GraphSAGE), giving the agent a richer understanding of audience sentiment, brand exposure, and stakeholder dynamics this explains higher engagement and sentiment accuracy. Third, the Meta-SAC algorithm enhances adaptability in dynamic sponsorship environments by continuously updating policies based on real-time feedback. Together, these mechanisms allow the agent to make informed, temporally coherent decisions rather than short-term reactive ones. Observed trade-offs, such as early exploration volatility and minor latency from multi modal processing, are acceptable given the substantial performance gains. Overall, the improvements are not incidental but arise directly from the framework’s hierarchical structure, multi-modal integration, and adaptive reinforcement learning design.

## Conclusion

This study demonstrates the effectiveness of using reinforcement learning in optimizing sports sponsorship through a multi-modal approach. Our system provides a dynamic, data-driven solution that significantly improves key metrics like ROI, brand exposure, and audience engagement. By leveraging RL in combination with CV, NLP, and GNNs, we introduce a scalable and adaptable framework that can be applied to real-world sports sponsorship management. Future work can explore integrating additional data sources and extending the framework to a wider range of sports and sponsorship contexts to further enhance its utility and performance.

## Data Availability

The datasets used in this study are publicly available and can be accessed through, Sports Event Dataset: https://www.kaggle.com/datasets/trolukovich/uiuc-sports-event-dataset Social Media Sentiments Analysis Dataset: https://www.kaggle.com/datasets/kashishparmar02/social-media-sentiments-analysis-datasetInteractions Dataset: https://products.monterosa.co/mic/integration-guide/audience-profiles/audience-profiles-dataset-reference/interactions-dataset.

## Code availability

The code for this study is available at: https://github.com/QiyangYu21/RL-Driven-Dynamic-Optimization-for-Sports-Sponsorship-Effectiveness-Evaluation.
